# Analyzing the Relationship Between Chronic Periodontitis and Psychological Stress in Elderly Patients: A Study Protocol

**DOI:** 10.7759/cureus.63869

**Published:** 2024-07-04

**Authors:** Tikeshwari Gurav, Priyanka Jaiswal, Deepika Masurkar

**Affiliations:** 1 Department of Periodontics, Sharad Pawar Dental College and Hospital, Datta Meghe Institute of Higher Education and Research, Wardha, IND

**Keywords:** lipp’s stress syndrome, old age home, stress, psychological issues, periodontitis

## Abstract

Background: Although stress is the body's physiological response to challenging life events, chronic exposure to a stressor may not cause stress. In such cases, stress negatively impacts some physiological functions, which can lead to psychosomatic diseases. According to research, inadequate coping mechanisms and long-term stress are factors that moderate the risk and progression of periodontitis. As a result, theories explaining how stress affects the periodontium have been put forth. The clinical examination comprised measurements of the probing pocket depth (PPD), clinical attachment level, number of teeth present, and plaque index. The current study uses a questionnaire to examine how psychosocial stress affects periodontium.

Methods: A written consent form will be obtained after each patient has received an extensive description of the study's objectives. The instrument for diagnosis and natural illumination will be used during the patient's examination. PPD and clinical attachment loss will be compared with a questionnaire.

Implications: Patients who are under stress should receive additional periodontal care to prevent periodontal disease from emerging or, if the issue already exists, from progressing to a more critical stage.

Conclusion: Stress here deserves special attention because it is a natural part of people’s life experiences with various intensities. Prejudice suggests that psychological stress and anxiety have a role in the advancement of periodontitis, which is consistent with the findings.

## Introduction

Bacteria, most of which are gram-negative, can cause the chronic illness known as periodontitis. The combination of oral and systemic microbiota conditions influences the severity of this disease. The periodontal ligament and the alveolar bone are particularly vulnerable to damage from this operation. There is evidence suggesting that immune system alterations related to anxiety and depression may increase the risk of periodontitis. In everyday life, negative events manifest as psychological stress and depression, emphasizing the person-environment relationship [1].

According to studies, individuals experiencing higher stress levels are more likely to have a more prevalent and severe form of periodontal disease [2]. There is a possible pathological concept for the role of stress in periodontitis [3]. This is because stress can weaken the immune system and lead to a change in behavior, encouraging harmful habits like smoking, binge drinking, and poor oral hygiene [4]. Psychological issues can cause patients to neglect oral hygiene, negatively affecting periodontal tissues [5].

In particular diseases such as acute necrotizing ulcerative gingivitis, there is a known association with depression, high levels of anxiety, and other emotional disorders. It is widely known that there is a connection between psychological risk factors and periodontal disease [6-8]. Changes in host defenses and health behaviors might result from social and environmental variables. The cause and mechanism of these variables' increased susceptibility to periodontal disease are unknown.

This study focuses on elderly patients residing in old age homes and how stress affects them. They faced many challenges, including a lack of knowledge of the medical condition, financial struggles, limited access to emergency services, and severe economic change. This period of their lives has proven to be the most stressful.

This study aimed to analyze the connection between elderly patients' psychological stress and chronic periodontitis, evaluate the periodontal status and clinical stress syndrome in elderly patients living in old age homes, and correlate the periodontal status with clinical stress syndrome in elderly patients living at old age homes. The implications are that patients who are under stress should receive additional periodontal care to prevent periodontal disease from emerging or, if the issue already exists, from progressing to a more critical stage.

## Materials and methods

Using the stress management questionnaire scale, stress and anxiety will be assessed by psychologists in individuals with varied degrees of chronic periodontitis. This age group will be chosen because patients staying in old age homes over the age of 40 are more likely to experience negative life events such as financial difficulties considering all-inclusive and exclusive factors, as shown in Table 1.

**Table 1:** Inclusion and exclusion criteria PPD: probing pocket depth; CAL: clinical attachment level; DM: diabetes mellitus; CVD: cardiovascular disease

Inclusion criteria	Exclusion criteria
The patients residing in nursing homes who are 40 years of age or older	Patients who had been taking immunosuppressive drugs
Patients having at least 20 teeth in their mouth	Immunocompromised individual
The patient's socioeconomic background and medical history	Individuals who had periodontal therapy six months before
Plaque index, gingival index, PPD, and CAL of the patients	Individuals having systemic diseases like DM, CVD, and respiratory disorders
Psychologists' approval regarding stress	Patients using tobacco products and smokers

All participants will be informed about the study's purpose before it begins, and their consent will be acquired. All participants will complete a questionnaire about demographics, socioeconomic status, health problems, history of health, and smoking.

Psychological assessment

Psychology students will utilize a created and validated psychological evaluation instrument, Lipp's stress syndrome for adults inventory [9], under the supervision of a psychologist to ascertain if a patient has clinical stress syndrome. The tool assists in determining the most obvious area of symptoms (physical or psychological). The tool also assists in evaluating stress symptoms across different time periods, helping to distinguish between acute and chronic stress, which can be crucial for developing targeted interventions. It also includes a thorough evaluation of the physical signs of stress, which are frequently disregarded by other screening methods.

Study method 2

Impartial examiners will conduct the clinical exams. William's periodontal probe will measure the probing pocket depth (PPD) and clinical attachment level (CAL) at six distinct locations on each tooth and report to the closest millimeter. It will be concluded that at least four teeth in those with a probing depth of more than or equal to 4 mm and a CAL of more than or equal to 3 mm have chronic localized periodontitis [10]. The plaque will be assessed using the Silness-Loe plaque index [11].

Although stress has been divided into three stages, a novel stage of the stress process known as near exhaustion has been discovered and statistically and clinically demonstrated because the term carries that name as it happens between resistance and exhaustion and is typified by a person's weakness in the face of an inability to resist or cope with the stressor. The psychologist will assess all the following symptoms, and then we will conduct a periodontal evaluation. The symptoms are examined in two categories: the exhaustion phase and the resistance phase, as shown in Table 2.

**Table 2:** Symptoms categorized in the exhaustion and resistance phases

Exhaustion phase	Resistance phase
Daily distress and worry	Memory problems
Nervousness	Ongoing fatigue
Severe fatigue	Appetite change
Inability to work	Hypertension
Apathy/disinterest, depression, or protracted rage	Queasy feeling
Irritability with no obvious reason	Appearance of skin problems
A diminished sense of humor	Unexplained generalized malaise
An eagerness to flee everything	Ulcer appearance
General sense of incapacity	Continuously feeling physically tired

Sample size estimation

The sample size will be estimated using the following formula:

η0 = (za + zβ)² (σ1²/r+σ20) / (d - δ)2

where the significance level type I error rate α = 0.05, power (1-beta) = 0.8, Z alpha value at 90% confidence = 1.645, Z beta value at 80% power = 0.842, ratio of sample size, treatment/control = 1, allowable difference (d) = μT − μC = 0, expected population standard deviation (SD) = 1.5, δ > 0 (margin) = 0.3, and dropout rate (%) = 8.

Result

The total sample size for the given study will be 200. Sample size will depend on the true mean difference, d, SDs for the two groups, a level of significance α (type | error), and the power. The total sample size n = n1 + n0 will be minimized when r = σ1/σ0. With the above-mentioned calculation, the sample size determination with a 90% confidence interval will be 200, considering the dropouts.

Statistical test

Demographic data will be analyzed using descriptive and frequency distributions. The impact of variables will be assessed using the chi-square test and Pearson correlational analysis. A linear regression model will help assess the relationship with demographic details.

Questionnaire

Table 3 displays the questionnaire, which contains ten questions that will be used to evaluate knowledge about stress. Each accurate response will receive a single score that ranges from 0 (no valid response) to 10 for each question (for all correct answers). A scoring system will be employed to determine the general degree of knowledge. Stress understanding will be graded as low (<3 points), moderate (>6 points), and severe according to the overall score (10 points). Participation in this study is entirely voluntary. The study's aim will be explained to each participant, and their privacy will be protected while their personal information is collected.

**Table 3:** Questionnaire to be given to the patients

S. no.	Questions
1	How frequently have you experienced distress due to an unforeseen event?
2	How frequently did you feel that you had no control over significant aspects of your life?
3	How frequently have you experienced anxiety and stress?
4	How frequently have you felt competent to deal with your own issues?
5	How frequently did you think things were going the way you wanted them to?
6	How frequently did you feel like you could not handle everything you had to?
7	How frequently have you been able to handle irritations in your life?
8	How frequently did you feel like you had everything under control?
9	How frequently have circumstances outside your control caused you to become enraged?
10	How many occasions have you thought that your problems were becoming too big for you to handle?

## Results

Of the total 200 participants, the stress score will be correlated with all three periodontal disease measures, i.e., gingival index, periodontal disease index, and bleeding on probing index. PPD and clinical attachment loss are predicted to rise in older adults residing in nursing homes in proportion to their stress levels compared to those living with their families. Participants' scores will vary depending on their knowledge of stress. The distribution of scores among participants may show a variety of scores indicating varying levels of stress knowledge. The scoring system creates clusters around the low, moderate, and severe classes. The study aims to shed light on participants' stress awareness and, based on their responses, categorize it as low, moderate, or severe. The results will aid in improving our understanding of how the general public views stress and could guide future initiatives in education and awareness.

## Discussion

The literature suggests that a stressful response to unfavorable socioeconomic circumstances can result in periodontal inflammation. Individuals experiencing psychological distress are more likely to develop chronic periodontitis than those who do not. Therefore, the destruction of periodontal tissues (gingiva, cement, periodontal ligament, and alveolar bone) and the emergence of periodontitis symptoms (gum bleeding, halitosis, clinical attachment loss, tooth mobility, and tooth loss) may be caused by immune system abnormalities that allow for increased pathogenicity of microorganisms [12,13]. However, periodontitis also results in an elevation of proinflammatory cytokines, which could heighten the inflammatory response throughout the body and serve as a risk factor for anxiety [14,15]. Additionally, a person's behavioral profile may have an impact on maintaining oral health through poor hygiene combined with dental care anxiety [16].

All the investigations in this area evaluated PPD and/or CAL to assess the periodontitis state [17]. In addition, other metrics like bleeding on probing (BOP) and gingival evaluation indexes were employed [17]. Using a mix of self-reported questionnaires and psychometric tests, anxiety levels were measured to determine the characteristics of the case group (periodontitis) and control group (no periodontitis). The state-trait anxiety inventory was also used by Solis et al. [18] to measure anxiety, but the findings did not reveal a significant correlation between periodontitis and anxiety. Castro et al. [17] postulated that periodontitis has a stronger direct correlation with sociocultural and demographic characteristics of the subject than it does with psychosocial factors to explain this result. Using the self-reported questionnaire, Solis et al. [18] addressed the possibility of individuals' emotional instability as one of the reasons for the lack of a link between periodontitis and anxiety since the responses depend on the subjective impression of the feelings themselves.

Based on the Newcastle Ottawa protocol, only the study by Laforgia et al. [19] had more methodological issues than the other studies that were part of this study. It was noted that confounding factor control was absent in the comparability domain. Consequently, even though the study found a link between periodontitis and anxiety, other characteristics, such as age and sex, may have also had an impact on the outcome. Small sample sizes and the use of self-reported questionnaires to diagnose anxiety are two common shortcomings in all the included research, which might lead to results that vary significantly depending on the participant's point of view at the time. Specifically, the study that found a link between anxiety and periodontitis did not look into the mechanics of this process.

Strengths of the study

Concentrating on elderly homes means that the research subject is more coherent and possesses similar age and similar living conditions in most cases, which can contribute to the decrease in the number of confounding factors associated with lifestyle and socioeconomic status. Chronic periodontitis is an easily recognizable clinico-morphological entity in which various treatment outcomes such as PPD, CAL, and bleeding on probing can be assessed. Evaluation of participants’ psychological stress using a standardized tool furnishes credible data on the psychological condition of the participants.

Limitations of the study

Stress measures using questionnaires may be influenced by the respondents’ perception and, therefore, may not reflect the ability of the individuals to handle stress. This leaves us with stress and periodontitis; however, social support, depression, and anxiety are interacting variables that could complicate the results. The studies carried out among the elderly homes may not reflect the findings of other elderly categories or independent elderly people.

## Conclusions

Stress here deserves special attention because it is a natural part of people’s life experience with various intensities. To improve the measurement of stress and its classification, new analytical tools similar to the Modified and Perceived Stress Scale questionnaires have been helped. These questionnaires do not contain sound psychological indicators or measurements and definitions of stress. If scientists were to administer more comprehensive questionnaires that encompass such factors in an individual’s life and other aspects such as gender, age, and character, then it would be possible to effectively determination of the level of psychological stress and the effect of this stress on an individual’s lifestyle. Prejudice suggests that psychological stress and anxiety have a role in the advancement of periodontitis, which is consistent with the findings.
